# Supplementary material for: Standardization of in situ coral bleaching measurements highlights the variability in responses across genera, morphologies, and regions
Source: PeerJ. 2023 Oct 2;11:e16100. doi: 10.7717/peerj.16100 (PMC10552771; doi:10.7717/peerj.16100)
Supplement: Supplemental Information 4 — Overview of each study’s (1) method of determining coral bleaching, (2) coral bleaching response metric, (3) level of taxonomic resolution, and (4) sampling time frame with regard to the bleaching event. [file peerj-11-16100-s004.docx]

**Supplementary Table 1:** Method of determining coral bleaching by study

| Method | References |
| --- | --- |
| ***In situ* surveys** | Bonilla 2001; Bruno et al. 2001; Clark et al. 2009; Couch et al. 2017; Dalton et al. 2020; Darling et al. 2013; Davies et al. 1997; Eriksson et al. 2012; Floros et al. 2004; Gleason 1993; Glynn et al. 2001; Guest et al. 2012; Guest et al. 2016; Hardman et al. 2007; Hussain & Ingole 2020; Ibrahim et al. 2017; Jiménez et al. 2001; Jokiel & Brown 2004; Kavousi et al. 2014; Kayanne et al. 2002; Kenyon & Brainard 2006; Kim et al. 2019; Knipp et al. 2020; Lang et al. 1992; Lenihan et al. 2008; Li et al. 2012; Loya et al. 2001; Marshall & Baird 2000; McClanahan 2004; McClanahan et al. 2005; McClanahan et al. 2007; McField 1999; Miller et al. 2011; Monroe et al. 2018; Montano et al. 2010; Muñiz-Castillo & Arias-González 2021; Obura 2001; Obura et al. 2018; Oxenford et al. 2008; Paulay & Benayahu 1999; Pengsakun et al. 2019; Quimpo et al. 2020; Rodgers et al. 2017; Sakai et al. 2019; Sebastián et al. 2009; Shuail et al. 2016; Stimson et al. 2002; Thinesh et al. 2019; Vargas -Ángel et al. 2011; Vo et al. 2020; Williams et al. 2010 |
| **Image analysis—** small scale (e.g., quadrats) | Carilli et al. 2017; Chou et al. 2016; Jones 2008; Kennedy et al. 2017; Matsuda et al. 2020; Ng et al. 2020; Valino et al. 2021; Van Woesik et al. 2012 |
| **Image analysis—** large scale (e.g., transects or mosaics) | Gintert et al. 2018; Johnston et al. 2019; Raymundo et al. 2019; Riegl et al. 2012; Teixeira et al. 2019; Tkachenko & Soong 2017 |
| **Video analysis** | Carroll et al. 2017; Frade et al. 2018; Nolan et al. 2021; Ritson-Williams & Gates 2020 |
| **Combination** of *in situ* surveys and analysis | Hédouin et al. 2020; Hoegh-Guldberg & Salvat 1995; Morgan et al. 2017; Porter et al. 2021; Spencer et al. 2000 |

**Supplementary Table 2:** Coral bleaching response metric by study

| Response metric | References |
| --- | --- |
| **Proportion of total colonies** completely or partially bleached, pale, and/or otherwise affected; “bleaching prevalence” | Carilli et al. 2017; Carroll et al. 2017; Chou et al. 2016; Clark et al. 2009; Couch et al. 2017; Davies et al. 1997; Floros et al. 2004; Frade et al. 2018; Gintert et al. 2018; Hardman et al. 2007; Hédouin et al. 2020; Hoegh-Guldberg & Salvat 1995; Hussain & Ingole 2020; Jiménez et al. 2001; Johnston et al. 2019; Jones 2008; Kavousi et al. 2014; Kayanne et al. 2002; Kennedy et al. 2017; Kenyon & Brainard 2006; Lenihan et al. 2008; Lang et al. 1992; Li et al. 2012; Marshall & Baird 2000; Matsuda et al. 2020; McField 1999; Miller et al. 2011; Monroe et al. 2018; Nolan et al. 2021; Obura 2001; Obura et al. 2018; Oxenford et al. 2008; Quimpo et al. 2020; Raymundo et al. 2019; Reyes-Bonilla 2001; Rodgers et al. 2017; Riegl et al. 2012; Ritson-Williams & Gates 2020; Sakai et al. 2019; Shuail et al. 2016; Teixeira et al. 2019; Thinesh et al. 2019; Van Woesik et al. 2012; Vargas-Ángel et al. 2011; Vo et al. 2020; Williams et al. 2010 |
| **Bleaching Index (BI)** developed by McClanahan; calculated as [*0c_1_+1c_2_+2c_3_+3c_4_+4c_5_+5c_6_*]*/5* where *c_1_* through *c_6_* are bleaching categories by colony | Dalton et al. 2020; Darling et al. 2013; Guest et al. 2012; Guest et al. 2016; Ibrahim et al. 2017; Kim et al. 2019; McClanahan 2004; McClanahan et al. 2005; McClanahan et al. 2007; Morgan et al. 2017; Muñiz-Castillo & Arias-González 2021; Ng et al. 2020; Pengsakun et al. 2019; Porter et al. 2021; Sebastián et al. 2009; Valino et al. 2021 |
| **Qualitative** (based on ﬁeld observations) | Bruno et al. 2001; Eriksson et al. 2012; Jokiel & Brown 2004; Loya et al. 2001; Paulay & Benayahu 1999; Spencer et al. 2000; Stimson et al. 2002; Tkachenko & Soong 2017 |
| **Within-colony** condition assessments for % pale, bleached, dead, etc. area | Gleason 1993; Glynn et al. 2001; Montano et al. 2010 |

**Supplementary Table 3:** Level of taxonomic resolution by study

| Level of resolution | References |
| --- | --- |
| **Genus-specific** | Carroll et al. 2017; Chou et al. 2016; Dalton et al. 2020; Darling et al. 2013; Davies et al. 1997; Eriksson et al. 2012; Floros et al. 2004; Frade et al. 2018; Gleason 1993; Guest et al. 2012; Guest et al. 2016; Hardman et al. 2007; Hédouin et al. 2020; Hoegh-Guldberg & Salvat 1995; Hussain & Ingole 2020; Ibrahim et al. 2017; Kavousi et al. 2014; Kayanne et al. 2002; Kennedy et al. 2017; Kim et al. 2019; Li et al. 2012; Marshall & Baird 2000; McClanahan 2004; McClanahan et al. 2005; McClanahan et al. 2007; Monroe et al. 2018; Montano et al. 2010; Morgan et al. 2017; Nolan et al. 2021; Obura et al. 2018; Porter et al. 2021; Quimpo et al. 2020; Sebastián et al. 2009; Shuail et al. 2016; Spencer et al. 2000; Teixeira et al. 2019; Thinesh et al. 2019; Tkachenko & Soong 2017; Valino et al. 2021; Van Woesik et al. 2012; Vargas-Ángel et al. 2011; Vo et al. 2020; Williams et al. 2010 |
| **Species-specific** | Bruno et al. 2001; Carilli et al. 2017; Clark et al. 2009; Couch et al. 2017; Gintert et al. 2018; Glynn et al. 2001; Jiménez et al. 2001; Johnston et al. 2019; Jokiel & Brown 2004; Jones 2008; Kenyon & Brainard 2006; Knipp et al. 2020; Lang et al. 1992; Lenihan et al. 2008; Loya et al. 2001; Matsuda et al. 2020; McField 1999; Miller et al. 2011; Muñiz-Castillo & Arias-González 2021; Ng et al. 2020; Obura 2001; Oxenford et al. 2008; Paulay & Benayahu 1999; Pengsakun et al. 2019; Raymundo et al. 2019; Reyes-Bonilla 2001; Riegl et al. 2012; Rodgers et al. 2017; Ritson-Williams & Gates 2020; Sakai et al. 2019; Stimson et al. 2002 |

**Supplementary Table 4:** Sampling time frame with regard to the bleaching event, by study

| Time of observation | References |
| --- | --- |
| **During** the event | Carilli et al. 2017; Chou et al. 2016; Frade et al. 2018; Glynn et al. 2001; Hédouin et al. 2020; Hoegh-Guldberg & Salvat 1995; Ibrahim et al. 2017; Jokiel & Brown 2004; Kavousi et al. 2014; Kenyon & Brainard 2006; Kim et al. 2019; Lenihan et al. 2008; McClanahan et al. 2007; Muñiz-Castillo & Arias-González 2021; Nolan et al. 2021; Paulay & Benayahu 1999; Pengsakun et al. 2019; Quimpo et al. 2020; Shuail et al. 2016; Teixeira et al. 2019; Valino et al. 2021; Van Woesik et al. 2012; Vo et al. 2020; Williams et al. 2010 |
| **Shortly after** (within weeks) | Couch et al. 2017; Gleason 1993; Guest et al. 2016; Hussain & Ingole 2020; Johnston et al. 2019; Jones 2008; Kennedy et al. 2017; Li et al. 2012; Marshall & Baird 2000; McClanahan et al. 2005; Monroe et al. 2018; Obura 2001; Oxenford et al. 2008; Rodgers et al. 2017; Riegl et al. 2012; Ritson-Williams & Gates 2020; Sebastián et al. 2009; Spencer et al. 2000; Tkachenko & Soong 2017; Vargas-Ángel et al. 2011 |
| **After** (>2 months later) | Bruno et al. 2001; Carroll et al. 2017; Davies et al. 1997; Eriksson et al. 2012; Lang et al. 1992; Obura et al. 2018; Raymundo et al. 2019; Reyes-Bonilla 2001; Sakai et al. 2019 |
| **Far after** (~6 months to one year later) | Guest et al. 2012; Miller et al. 2011; Stimson et al. 2002 |
| **Continual** (before, during, and/or after) | Clark et al. 2009; Dalton et al. 2020; Darling et al. 2013; Floros et al. 2004; Gintert et al. 2018; Hardman et al. 2007; Jiménez et al. 2001; Kayanne et al. 2002; Knipp et al. 2020; Loya et al. 2001; Matsuda et al. 2020; McClanahan 2004; McField 1999; Montano et al. 2010; Morgan et al. 2017; Ng et al. 2020; Porter et al. 2021; Thinesh et al. 2019 |
